# The Early Microbial Colonizers of a Short-Lived Volcanic Island in the Kingdom of Tonga

**DOI:** 10.1128/mbio.03313-22

**Published:** 2023-01-11

**Authors:** Nicholas B. Dragone, Kerry Whittaker, Olivia M. Lord, Emily A. Burke, Helen Dufel, Emily Hite, Farley Miller, Gabrielle Page, Dan Slayback, Noah Fierer

**Affiliations:** a Department of Ecology and Evolutionary Biology, University of Colorado Boulder, Boulder, Colorado, USA; b Cooperative Institute for Research in Environmental Science, Boulder, Colorado, USA; c Corning School of Ocean Sciences, Maine Maritime Academy, Castine, Maine, USA; d Sea Education Association, Woods Hole, Massachusetts, USA; e School of Marine Science and Ocean Engineering, University of New Hampshire, Durham, New Hampshire, USA; f Scripps Institute of Oceanography, University of California San Diego, La Jolla, California, USA; g School of Earth Sciences and Environmental Sustainability, Northern Arizona University, Flagstaff, Arizona, USA; h Université de Bretagne Occidentale, Brest, Brittany, France; i Science Systems & Applications, Inc, Lanham, Maryland, USA; j Biospheric Sciences Lab, NASA Goddard Spaceflight Center, Greenbelt, Maryland, USA; University of California, Irvine

**Keywords:** volcanoes, Tonga, microbial ecology

## Abstract

The island of Hunga Tonga Hunga Ha’apai (HTHH) in the Kingdom of Tonga was formed by Surtseyan eruptions and persisted for 7 years before being obliterated by a massive volcanic eruption on 15 January 2022. Before it was destroyed, HTHH was an unparalleled natural laboratory to study primary succession on a newly formed landmass. We characterized the microbial communities found on the surface sediments of HTHH using a combination of quantitative PCR, marker gene sequencing, and shotgun metagenomic analyses. Contrary to expectations, photosynthetic cyanobacteria were not detected in these sediments, even though they are typically dominant in the earliest stages of primary succession in other terrestrial environments. Instead, our results suggest that the early sediment communities were composed of a diverse array of bacterial taxa, including trace gas oxidizers, anoxygenic photosynthesizers, and chemolithotrophs capable of metabolizing inorganic sulfur, with these bacteria likely sourced from nearby active geothermal environments. While the destruction of HTHH makes it impossible to revisit the site to conduct *in situ* metabolic measurements or observe how the microbial communities might have continued to change over time, our results do suggest that the early microbial colonizers have unique origins and metabolic capabilities.

## INTRODUCTION

Microorganisms are often the earliest colonizers of newly exposed or newly formed terrestrial surfaces (1) and have important roles in the development of ecosystems and the subsequent process of community succession (2). In such environments, like a freshly cleaved rock face (3, 4), recently deposited volcanic ash (5, 6), or sediments exposed following glacial retreat (7, 8), these early microbial colonizers tend to include oligotrophs, autotrophs, and other taxonomic groups that can survive in nutrient-limited environments and/or fix carbon and nitrogen (1, 9, 10). However, the identities of these earliest colonizers are likely dependent on the specific environment in question. For example, while photosynthetic cyanobacteria are often the earliest colonizers of sediments exposed after glacial retreat or newly formed sand dunes (7, 11, 12), the microbial colonizers of lava flows can also include many chemolithotrophs (13).

One of the most dramatic examples of microbial colonization occurs after the creation of new land. Most often. this is a result of a volcanic eruption covering an existing surface with lava, ash, or tephra (5, 14). On occasion, volcanoes can also form completely new landmasses. “Surtseyan” eruptions take place in shallow waters and rapidly push ejecta up through the water column (15, 16), often leading to the formation of new islands (17–19). While many such Surtseyan islands rapidly erode (within months to a few years) (20, 21), some may persist for long enough to be colonized by organisms (22, 23). A unique aspect of newly formed volcanic islands is the “blank slate” they provide. Like other newly exposed terrestrial surfaces, there is initially little, if any, organic carbon on the sediments of new volcanic islands (24–26). Such volcanic substrates can also be challenging environments for organisms to recruit to and survive in because they typically contain high concentrations of heavy metals and are exposed to toxic volcanic gases, though the exact physical and chemical properties of these systems can vary depending on the geologic context (6, 13, 27, 28).

When the last two persisting Surtseyan islands formed in the 1950s (Capelinhos, Azores) and 1960s (Surtsey, Iceland), research on these islands primarily focused on animal and plant colonizers, not microorganisms (22, 23). For example, while qualitative observations of bacteria and algae were recorded as early as 1966 on Surtsey (22, 29), the namesake of the eruption type, they were considered “extremely minor in importance” (22). In fact, the first comprehensive survey of microorganisms on a Surtseyan island did not occur until 2000, almost 40 years after the island of Surtsey had formed (30). Thus, we have limited knowledge of the earliest microbial colonizers on newly formed volcanic islands. However, from work conducted in other volcanic systems, primary successional systems, and recent boreholes drilled on Surtsey (31, 32), we can hypothesize that the initial microbial colonizers likely include autotrophic taxa able to fix carbon by using light energy (phototrophs) or taxa that use inorganic energy sources to build biomass (chemolithotrophs) via trace gas oxidation, sulfur oxidation, and/or iron oxidation (13, 27, 33, 34). The earliest microbial colonizers may also include oligotrophic heterotrophs able to survive on trace amounts of C and N in the island’s sediments or deposited by dust, sea spray, or from the atmosphere (35, 36).

On 19 December 2014, an underwater volcano in the Kingdom of Tonga in the southwestern Pacific Ocean began a month-long series of eruptions. A new island, referred to as Hunga Tonga Hunga Ha’apai (HTHH), had emerged by the end of January 2015 (37, 38). This new landmass formed between the two older islands of Hunga Tonga (HT) and Hunga Ha’apai (HH), connecting them with an ~120-m tall, ~1.9-km^2^ tuff cone of tephra and ash. HTHH, the subaerial projection of the much larger Hunga volcano, was initially expected to erode after a few months but, instead, persisted (37). HTHH is only the third such landmass to have formed in the past 150 years that has persisted for longer than a year (38). Unlike Capelinhos and Surtsey that preceded it, HTHH formed in the tropics (latitude, 20.5°S). While the tuff cone that formed in 2015 was destroyed during the explosive eruption of the Hunga volcano in 2022, which also stripped HT and HH of tens of meters of rock and sediment (39), samples of tephra collected from across the island 3 and 4 years after formation offered a rare opportunity to study the early microbial colonizers of the island’s sediments. Using a suite of microbiological approaches, including cultivation-independent marker gene sequencing, metagenomic “shotgun” sequencing, and quantitative PCR combined with a range of geochemical analyses, we addressed three questions. What taxa are the earliest microbial colonizers of sediments on HTHH? From where did these microbial colonizers originate? What metabolic strategies are used by these microbes to persist in the challenging environmental conditions found on the recently formed landmass?

## RESULTS AND DISCUSSION

**FIG 1:**
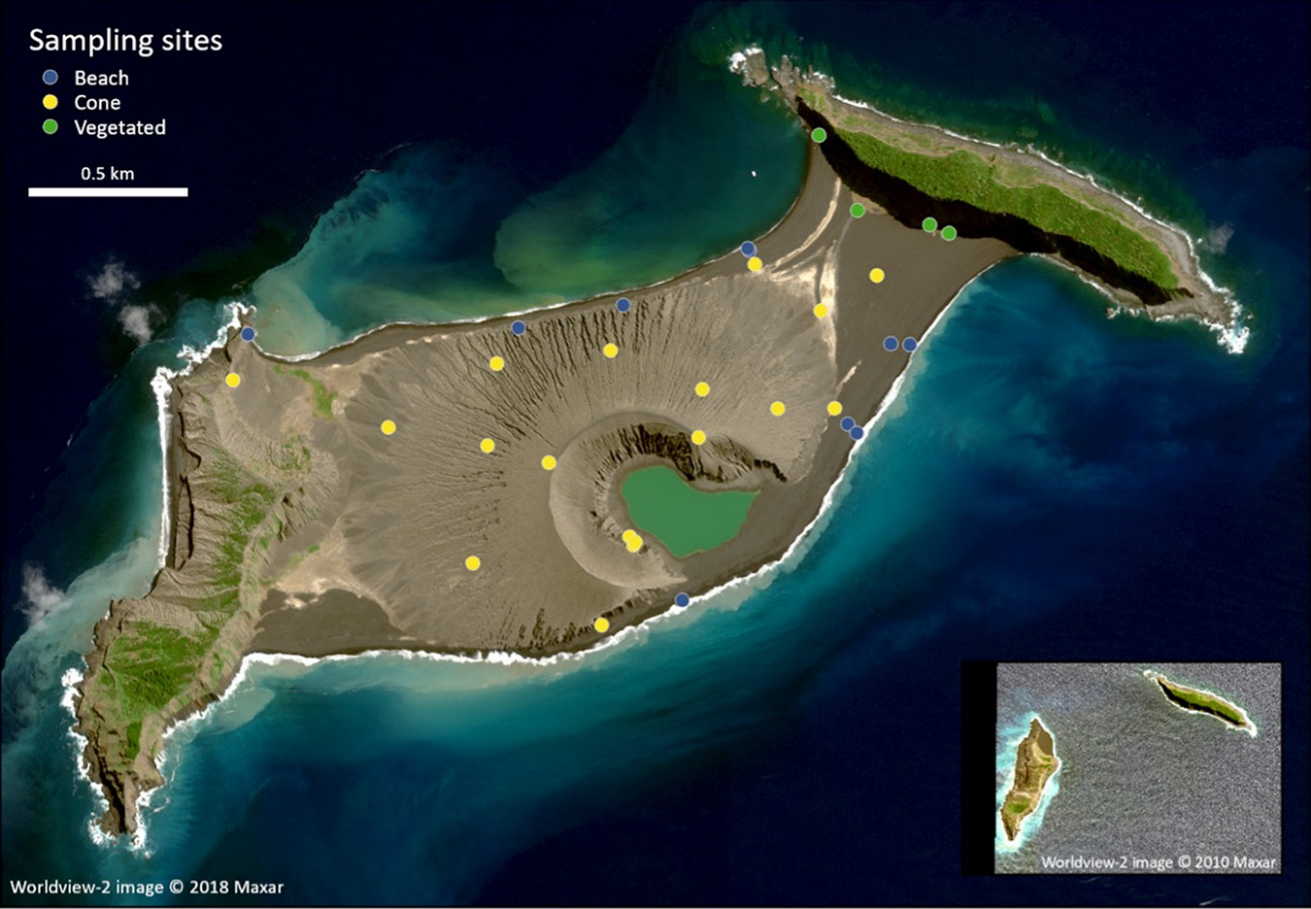
The island of Hunga Tonga Hunga Ha’apai, Kingdom of Tonga (latitude, 20.536°S; longitude, 175.382°W). The locations of the 32 surfaces where samples were collected are shown. The background image is from 19 August 2018 and is orthorectified. The inset image displays the islands of Hunga Ha’apai (west) and Hunga Tonga (east) on 11 September 2010, prior to the 2014–2015 eruption. Worldview-2 image © 2010, 2018 Maxar.

We collected 32 samples across the 1.9-km^2^ volcanic cone of HTHH from surfaces that ranged from sea level to the summit of the crater ~120 m above sea level (masl). While the focus of this study was on the unvegetated surfaces of the island cone, samples were also collected from sediments at the beach (marine-terrestrial interface) and from vegetated sediments around the island of HT that predate the 2014–2015 eruption cycle (Fig. 1). While plants and animals could be found on the island at the time of collection, samples were collected from surfaces that were not visibly colonized by plants or animals unless otherwise noted (see Fig. 1, “vegetated” samples). For information on terrestrial and aquatic macrofauna and flora on HTHH, see references 38 and 40.

The properties of the unvegetated inland sediments collected from the tuff cone of HTHH suggest a distinct environment characterized by low concentrations of nutrients and organic carbon, high metal concentrations (including potentially toxic metals), and appreciable levels of sulfur (Fig. S1 and Data Set S1A in the supplemental material). Organic carbon concentrations in the cone sediments were low (average, 0.32 mg.g^−1^; range, 0.19 to 0.50 mg.g^−1^) and typically 10 times lower than organic carbon concentrations in the vegetated samples, which were collected from sediments where plants had recruited near the edge of HT (Fig. 1; Fig. S1). The cone sediments had no detectable nitrogen but high concentrations of sulfur (average, 2.1 mg.g^−1^; range, 0.1 to 19.8 mg.g^−1^), and iron (average, 801 mg.g^−1^; range, 74 to 86 mg.g^−1^) (Fig. S1; Data Set S1A). The cone sediments also had high concentrations of other metals (Data Set S1A), including copper, vanadium, and cobalt concentrations that exceed those typically found in natural soils and are similar to the concentrations often found in metal-contaminated industrial sites and other volcanic systems (Fig. S1) (41–44).

10.1128/mbio.03313-22.1 FIG S1 Environmental and geochemical characteristics of the samples collected from across the island of HTHH. The pH of the inland cone sediments (*n* = 13) was significantly lower than the pH of the beach sediments (*n* = 10) or the vegetated sediments (*n* = 4) (Kruskal-Wallis one-way analysis of variance, *P* < 0.001). Total organic carbon in the vegetated samples was significantly higher than in the other sample categories (Kruskal-Wallis, *P* < 0.001), and sulfur content (%) was significantly higher in the cone samples than in the vegetated samples (Mann-Whitney U test, *P* = 0.033). Sediments from both cone and vegetated sediments had high concentrations of copper, vanadium, cobalt, and scandium, with these concentrations well above the range typically measured in “typical,” noncontaminated soils (indicated in red, taken from references [Bibr B41][Bibr B42][Bibr B44]). Download FIG S1, TIF file, 0.6 MB. Copyright © 2023 Dragone et al. 2023 Dragone et al. https://creativecommons.org/licenses/by/4.0/ This content is distributed under the terms of the Creative Commons Attribution 4.0 International license.

10.1128/mbio.03313-22.10 DATA SET S1 (A) Location and environmental/geochemical characteristics associated with the samples collected from HTHH in 2018 and 2019 (see Materials and Methods for more details). Values that were not recorded for a sample are noted as “NA.” (B) Table of all ASVs recovered from amplicon sequencing of the 16S rRNA gene with at least 10 reads in total across the entire dataset (4,456 ASVs) for the 27 samples that passed our threshold of inclusion (see Materials and Methods for more details). The relative abundance of each ASV in each sample is represented as the proportion of total reads assigned to that sample. Taxonomic classification of each ASV and the 16S rRNA sequence for that ASV are also included. (C) Information about the 156 metagenomic-assembled genomes (MAGs) recovered from the inland cone samples (see Materials and Methods for more details). Other information, including the sample the MAG was recovered from, the identity of each MAG, quality metrics, and the closest placement reference if one was found, is also listed. Also included are the number of reads identified in these samples associated with genes related to CO oxidation, H_2_ oxidation, anoxygenic photosynthesis, and thiosulfate transformations in sulfur metabolism. (D) Table of the normalized abundance of the 294 genes associated with chemolithotrophic pathways generated through the targeted gene analysis. The abundance of each gene is normalized based on the abundance of 14 single-copy genes and is presented as normalized reads per million (RPM) (see Materials and Methods for more details). Download Data Set S1, XLSX file, 1.0 MB. Copyright © 2023 Dragone et al. 2023 Dragone et al. https://creativecommons.org/licenses/by/4.0/ This content is distributed under the terms of the Creative Commons Attribution 4.0 International license.

### Bacterial and archaeal communities on HTHH.

**FIG 2:**
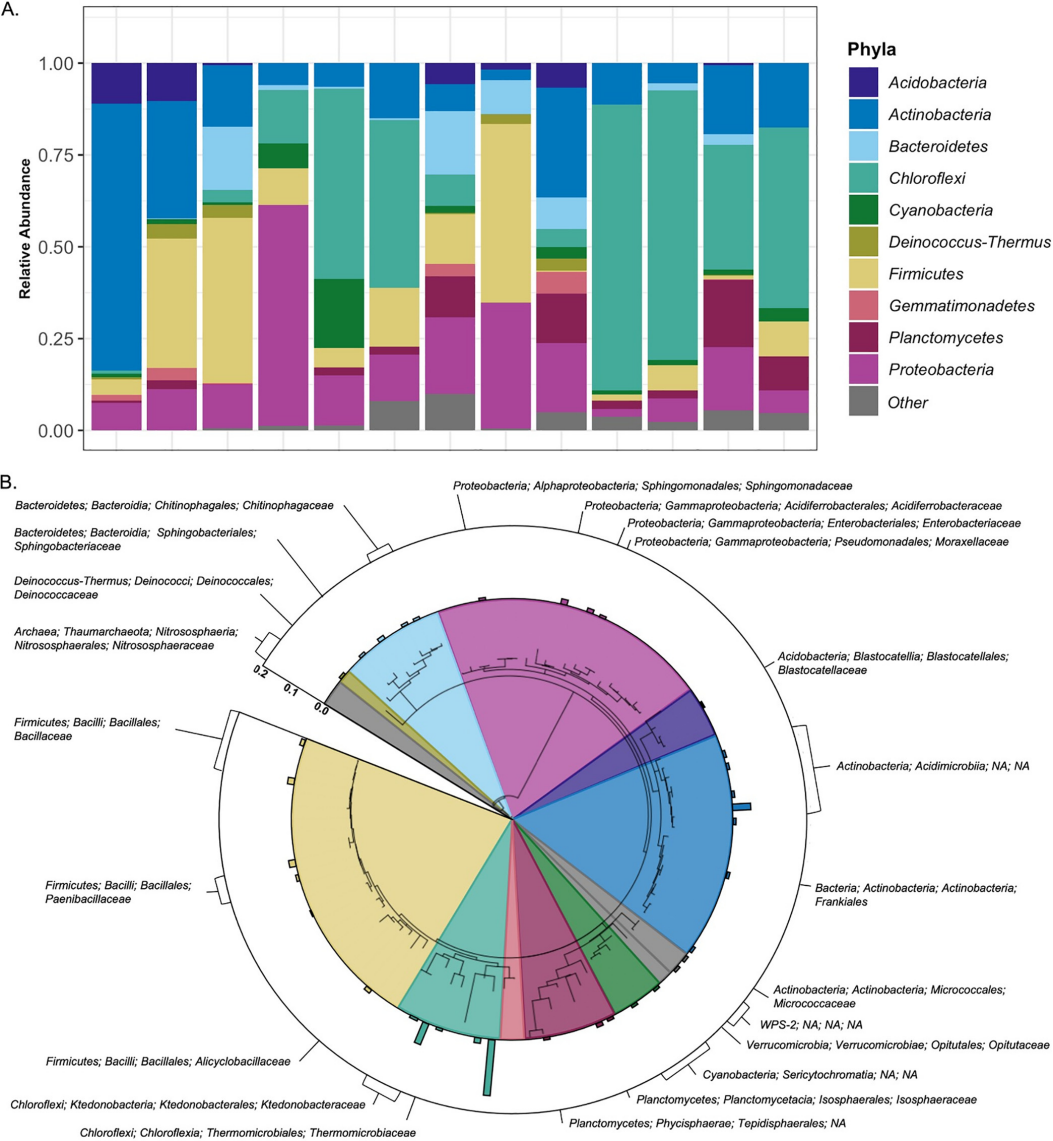
Overview of the microbial community composition in the inland cone sediments. (A) Proportional abundances of the dominant prokaryotic phyla found in each of the 13 inland cone samples from which 16S rRNA gene sequences were obtained. (B) Phylogenetic tree of the most abundant amplicon sequence variants (ASVs) identified in the inland cone samples of HTHH. The ASVs represented in this tree include the 100 most abundant prokaryotic ASVs that were identified from the 16S rRNA gene amplicon sequencing (2 archaeal sequences, 98 bacterial sequences). The inset colors indicate the region of the tree associated with each bacterial phylum, and the proportional abundances of each ASV across the whole sample set are represented with the exterior bar plot. If an ASV made up less than 0.05% of reads, no bar is displayed. For those bacterial families that passed this abundance threshold, taxonomic information (phyla, class, order, family) is displayed outside the bar plot. If an ASV could not be classified past the phylum level of resolution, no taxonomic label is included.

Bacteria and archaea were detected in all the cone samples, but the amount of prokaryotic DNA, as determined via quantitative PCR, was 2 orders of magnitude lower in the cone samples than in the vegetated sediments collected around the island of HT (Fig. S2). The cone prokaryotic communities were also less diverse, with a mean of 108 amplicon sequence variants (ASVs) detected via targeted 16S rRNA gene sequencing compared to a mean of 473 ASVs in the vegetated samples (Fig. S3). The prokaryotic communities found in the unvegetated cone samples were also distinct in composition (Fig. 2, Fig. S4, and Data Set S1B) and dominated by members of the bacterial phylum *Chloroflexi*, which made up 26.4% of reads associated with these samples. *Actinobacteria* (18.1% of total reads), *Firmicutes* (15.7%), and *Proteobacteria* (15.5%) were also abundant. Other phyla that were identified in these samples included *Bacteroidetes* (6.2%), *Planctomycetes* (5.4%), *Acidobacteria* (3.3%), *Cyanobacteria* (2.8%), *Gemmatimonadetes* (1.5%), and candidate phylum WPS-2 (“Candidatus *Eremiobacteria*,” 1.5%). Archaea belonging to the phylum *Thaumarchaeota* were found in all samples, but they were relatively rare (<2% of reads across all samples). These taxonomic results obtained from targeted 16S rRNA gene sequencing mirrored results obtained by conducting shotgun metagenomic analyses on a subset of the same samples (Fig. S5). The dominant bacterial families identified in the cone samples included *Acidiferrobacteraceae*, *Ktedonobacteraceae*, and *Sulfuricellaceae*, which include organisms that have been classified as autotrophic chemolithotrophs capable of oxidizing sulfur and iron based on studies conducted in other systems (45, 46). However, we note that many of the taxa come from groups that are poorly characterized. Of the top 100 taxa recovered with our targeted 16S rRNA gene sequencing, 40% could not be classified to a bacterial family (Fig. 2). The early arrivals to the sediments of this new land mass are predominately bacterial taxa for which preexisting information on their ecologies is limited; only 52% of the top 100 ASVs were from families that have previously been cultivated, thus necessitating the metagenomic-based analyses detailed below.

10.1128/mbio.03313-22.2 FIG S2 Amount of prokaryotic (bacterial and archaeal) DNA found in the sediments of HTHH, as estimated by quantitative PCR (qPCR) analysis of the 16S rRNA gene. Cone sediments (*n* = 13) had significantly lower DNA concentrations than the beach sediments (*n* = 10) and vegetated sediments (*n* = 4) (Kruskal-Wallis one-way analysis of variance, *P* < 0.001). *Post hoc* comparisons between groups, indicated with letters above each group, were performed using Dunn’s multiple-comparison test. Download FIG S2, TIF file, 2.1 MB. Copyright © 2023 Dragone et al. 2023 Dragone et al. https://creativecommons.org/licenses/by/4.0/ This content is distributed under the terms of the Creative Commons Attribution 4.0 International license.

10.1128/mbio.03313-22.3 FIG S3 Prokaryotic richness (number of distinct prokaryotic ASVs out of 10,000 reads per sample) of the beach, cone, and vegetated sediments. Cone sediments (*n* = 13) had significantly lower prokaryotic richness concentrations than the beach sediments (*n* = 10) and vegetated sediments (*n* = 4) (Kruskal-Wallis one-way analysis of variance, *P* < 0.001). *Post hoc* comparisons between groups, indicated with letters above each group, were performed using Dunn’s multiple-comparison test. Download FIG S3, TIF file, 2.1 MB. Copyright © 2023 Dragone et al. 2023 Dragone et al. https://creativecommons.org/licenses/by/4.0/ This content is distributed under the terms of the Creative Commons Attribution 4.0 International license.

10.1128/mbio.03313-22.4 FIG S4 Overview of bacterial and archaeal community composition across the island of HTHH. Shown here are the relative abundances of the top 10 most abundant phyla for each of the 27 samples for which 16S rRNA marker gene sequence data were obtained. Samples are grouped by environment type (cone, beach, vegetated). Download FIG S4, TIF file, 2.5 MB. Copyright © 2023 Dragone et al. 2023 Dragone et al. https://creativecommons.org/licenses/by/4.0/ This content is distributed under the terms of the Creative Commons Attribution 4.0 International license.

10.1128/mbio.03313-22.5 FIG S5 Taxonomic information recovered from the amplicon and shotgun metagenomics sequencing efforts for the cone (*n* = 13) and vegetated sediments (*n* = 4). (A) The relative abundances of the top 10 most abundant phyla as measured by amplicon sequencing of the 16S rRNA gene. (B) Relative abundance of the top 10 most abundant phyla based on the 16S rRNA gene sequences recovered from the metagenomic sequencing data with phyloFlash (87). We also note that the taxonomic composition of the prokaryotic communities, as inferred from the 16S rRNA amplicon data, was well correlated with the taxonomic composition inferred from the metagenomic data (Mantel test of Bray-Curtis dissimilarity matrices, *r* = 0.90, *P* < 0.001). Download FIG S5, TIF file, 2.0 MB. Copyright © 2023 Dragone et al. 2023 Dragone et al. https://creativecommons.org/licenses/by/4.0/ This content is distributed under the terms of the Creative Commons Attribution 4.0 International license.

In contrast to the prokaryotic taxa commonly found in other newly exposed terrestrial surfaces, including surfaces exposed after glacial retreat (1, 10), the HTHH communities are distinct. For example, photosynthetic cyanobacteria, which are often considered to be characteristic of the earliest microbial communities (7), are completely absent, with the only cyanobacteria identified across all samples associated with the nonphotosynthetic lineage of “*Candidatus* Sericytochromatia” (17, 47). We expect that the absence of cyanobacteria is due to the high concentrations of hydrogen sulfide (H_2_S) typically released during volcanic activity, as H_2_S has been shown to restrict the growth of cyanobacteria and other oxygenic phototrophs (48). However, we did find that members of the *Chloroflexi* phylum dominated the microbial communities in the HTHH sediments (Fig. 2; Data Set S1B). While this phylum does not typically represent such large a proportion of the communities found in other early successional terrestrial environments (11), it is often found in volcanic environments where H_2_S is present (13, 27, 28).

The cone samples are generally similar to those identified in other microbial communities described in studies of older volcanic deposits (sampled >10 years posteruption) (13, 27, 34, 49). To highlight one example, a study of basaltic lava deposits in Iceland found that the orders *Planctomycetales*, *Rhizobiales*, *Rhodospirillales*, and *Sphingomonadales* were ubiquitous and abundant in all sampled basalts (50). These orders were also dominant in the cone samples (Fig. 2; Data Set S1B). Likewise, representatives of the phylum *Chloroflexi*, which were particularly abundant on HTHH, were also ubiquitous across Icelandic basalts (50).

### Source of bacterial and archaeal diversity.

What are the likely sources of those microbes found to be dominant in the HTHH cone sediments? We might expect many of these microbes to be derived from surrounding ocean waters or from birds that deposit gut bacteria on the newly formed land mass. This does not seem to be the case. The bacterial taxa commonly associated with the bird microbiome and the taxa most often found in the marine environment were rare in the cone sediments (Table S1) (51, 52), though we expect bird gut bacteria may be more abundant near nesting sites that were not sampled. Alternatively, the microbes we have classified on HTHH may have dispersed onto the cone from the soils found on the flanking islands of HT and HH that predated the 2014–2015 eruption, either blown by the wind or carried by plants as they spread across the land bridge (38). While the microbial communities that dominate the vegetated sediments from HT (Fig. 1) do share taxa with those in the HTHH cone samples (Fig. S6), our data do not suggest that these, or other, soils are the main source of microbes found in the cone sediments. Of the top 100 ASVs found in the cone samples, which make up, on average, 77% of the total reads in these samples (50 to 94% of reads), only 58 ASVs can be found in the vegetated samples, and they are generally rare, representing only 23% of the total reads in the vegetated samples on average (6 to 57% of reads) (Fig. S7). Likewise, if we directly compare the ASVs from the cone samples to the 511 ASVs previously reported as being abundant and nearly ubiquitous in soils worldwide (53), we find that only 19 of the cone ASVs match those in this reference database. In fact, only 19% of all reads from the cone sediments were assigned to bacterial classes that are the most common in soils (see reference 53), compared to 43% of all reads in the vegetated samples (Table S1). While we cannot exclude the possibility that some of the cone microbes are derived from neighboring soils (or vice versa), the minimal overlap between the cone communities and those found on the neighboring islands or in a comprehensive database of global soils (53) suggests that most of the microbial colonizers on HTHH are unlikely to originate from soil.

10.1128/mbio.03313-22.6 FIG S6 Shared prokaryotic taxa across the different sediment environments. The Venn diagram was created with the ASV list compiled from the 16S rRNA gene sequencing effort of the beach (*n* = 10), cone (*n* = 13), and vegetated sediments (*n* = 4). Download FIG S6, TIF file, 1.6 MB. Copyright © 2023 Dragone et al. 2023 Dragone et al. https://creativecommons.org/licenses/by/4.0/ This content is distributed under the terms of the Creative Commons Attribution 4.0 International license.

10.1128/mbio.03313-22.7 FIG S7 Variation in microbial community composition across the island of HTHH. (A) Nonmetric multidimensional scaling (NMDS) plot based on the Jaccard distances in the 27 samples analyzed via 16S rRNA gene sequencing with colors indicating each of the three environment types sampled. (B) The proportional abundances of the top 100 ASVs identified from the cone samples in each of the three other environments that were sampled. Samples are arranged by environment (indicated above the stacked bars), and ASVs are colored by phyla. Download FIG S7, TIF file, 2.3 MB. Copyright © 2023 Dragone et al. 2023 Dragone et al. https://creativecommons.org/licenses/by/4.0/ This content is distributed under the terms of the Creative Commons Attribution 4.0 International license.

10.1128/mbio.03313-22.8 TABLE S1 Potential sources of microbial colonizers. (A) Abundance of the most ubiquitous bacterial families found in bird guts (as defined by reference 52) in the inland cone samples. (B) Abundance of the most common bacterial classes found in the marine environment (based on reference 51) in the inland cone samples. (C) Abundance of the dominant bacterial phylotypes found in soils. Percentage of total reads in the inland cone samples and the vegetated samples that are associated with the most common bacterial phylotypes found in soils, as defined by reference 53. Download Table S1, TIF file, 1.8 MB. Copyright © 2023 Dragone et al. 2023 Dragone et al. https://creativecommons.org/licenses/by/4.0/ This content is distributed under the terms of the Creative Commons Attribution 4.0 International license.

Instead, we hypothesize that the microbial taxa found in the cone sediments may have been sourced from nearby volcanic systems and/or hydrothermal systems. While no detailed microbial information or equivalent sequence data are available from Tonga’s active geothermal systems or from the seafloor and subsurface around HTHH pre-eruption, the communities we identified in our samples share similar taxa to those often found in these types of environments. Many of the most abundant sequences we identified, including those assigned to *Planctomycetales*, *Rhizobiales*, *Rhodospirillales*, and *Sphingomonadales*, are most similar to sequences in reference databases recovered in studies of volcanic environments in Iceland, Hawaii, New Zealand, and Alaska (34, 49, 50, 54). More specifically, we note that some of the more abundant taxa in the cone samples, including the uncultivated *Chloroflexi*, which dominate our communities, are typically well represented in deep euphotic-zone water (55), around hydrothermal vents (56), and organic-poor seafloor and subsurface sediments (57). In fact, the most abundant 16S rRNA gene sequence found in the cone samples (ASV_1, *Bacteria*, *Chloroflexi*, AD3) is an exact sequence match to *Chloroflexi* recovered from sediments of the Brothers Volcano Complex in the Tonga-Kermadec Arc (58), along with 12 of the other most abundant sequence variants. Finally, we also find that several of the more dominant taxa in the cone samples represented in our collection of metagenome-assembled genomes (MAGs) are most similar to MAGs obtained from Yellowstone hot springs (46) and hydrothermal vent fields in the Atlantic and Pacific (Data Set S1C) (56, 59).

The island of HTHH was simply the subaerial projection of the much larger submarine Hunga volcano, whose underwater caldera covers a total area of ~16 km^2^. This volcano is extremely active, with large eruptions in 2009, 2014 to 2015, and 2022 following submarine and subaquatic venting and activity recorded since 1912 (19, 37, 39, 60). We see two potential mechanisms that could have brought organisms from the subsea or subsurface to the subaerial cone. First, organisms found in the sediments around the active crater may have been transported up from the seafloor and subsurface sediments during the 2014–2015 eruption event that formed the island. Vertical microbial transport like this has been described on Surtsey where microbes found in the subsurface pore water and sediments, which includes *Chloroflexi*, were shown to be brought to the surface by fumarolic activity (32). It is also possible that organisms may have been blown in from nearby subaerial volcanic systems. HTHH is just one of ~20 active volcanoes in Tonga. The nearest, Fonuafo’ou, although submarine, is only 25 km away, and Tofua, which has frequent fumarolic activity, is 100 km away (60). Nearby explosive activity, like that of Late’iki in 2019 (61), may have aerosolized and transported volcanic-associated microbes. From work conducted in New Zealand, we know that such eruption-mediated microbial dispersal can transport viable microbial cells over distances exceeding 850 km (62). However, due to the presence of the sediment-associated volcanic ASVs (described previously), we would expect that in the case of HTHH, microbial transport from the subsurface is a more likely explanation.

### Inferred metabolic strategies of the microbial colonizers.

Based on taxonomic information alone, we hypothesized that the microbial communities found on the newly formed land mass are dominated by chemolithotrophic bacteria and anoxygenic phototrophs. To infer the metabolic strategies used by these communities, we conducted shotgun metagenomic sequencing on a subset of samples, which included all the unvegetated, inland sediments collected from the cone (*n* = 13), with four vegetated samples included for comparison. We analyzed these metagenomic data sets to quantify the abundances of reads in each sample assigned to ~300 genes (Data Set S1D) associated with metabolic strategies that we expect to be used by microbes to persist in this and other volcanic systems (13, 34). We found that genes associated with pathways involving sulfur metabolism, CO oxidation, H_2_ oxidation, and bacteriochlorophyll-mediated anoxygenic photosynthesis were enriched in the cone samples with the normalized abundance of genes associated with these pathways ~2 to 5 times higher than in the vegetated samples (Table S2). For information on the relative abundances of genes associated with other functions, none of which were significantly different between the two categories of samples, see Table S2 and Data Set S1D.

10.1128/mbio.03313-22.9 TABLE S2 Abundance of the genes related to 20 metabolic pathways in the cone (*n* = 13) and vegetated samples (*n* = 4). The abundances of genes are presented as the average normalized reads per million (RPM) of all samples in that group. The significance (*P* value) was determined by a Mann-Whitney U test. Genes are grouped based into pathways based on classifications given in references 59, 63, and [Bibr B89][Bibr B90][Bibr B91], with each gene appearing in just one group based on what function they have most often been described in. However, we note that many of these genes may be utilized by multiple pathways (for example, genes shared by sulfur reduction and sulfur oxidation). Data on individual gene abundances can be found in Dataset S1C. Download Table S2, TIF file, 1.8 MB. Copyright © 2023 Dragone et al. 2023 Dragone et al. https://creativecommons.org/licenses/by/4.0/ This content is distributed under the terms of the Creative Commons Attribution 4.0 International license.

**FIG 3:**
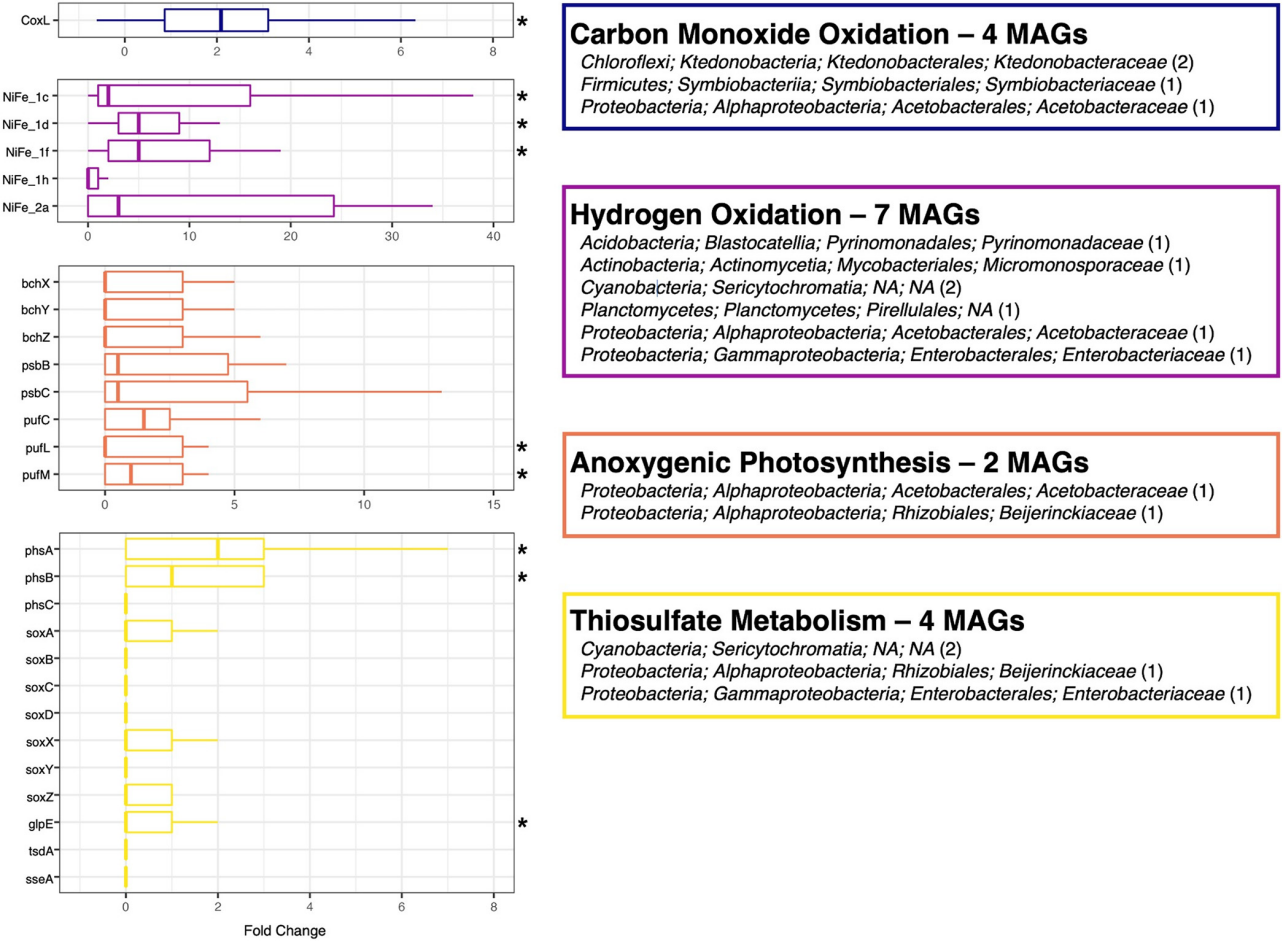
Differential abundances of genes between the inland cone sediments (*n* = 13) and the vegetated sediments (*n* = 4). The 27 genes are associated with the metabolic pathways of thiosulfate transformations in sulfur metabolism (13 genes), anoxygenic photosynthesis (8 genes), hydrogen oxidation (5 genes), and carbon monoxide oxidation (1 gene). Abundances are presented as the fold change in gene abundances (normalized reads per million) compared to the average gene abundances in the vegetated samples. Genes with significantly higher abundances in cone samples are indicated by a star (Mann-Whitney U test, *P* < 0.05). The number of MAGs containing the genes associated with these four metabolic categories is displayed in the boxes on the right with the taxonomy, and the number of MAGs within each taxonomic group is indicated. If taxonomy could not be resolved below a certain level, it is listed as “NA.”

Our finding that genes associated with sulfur metabolism are abundant in the HTHH sediment communities is in line with the high concentrations of sulfur measured in these samples (Data Set S1A). A closer investigation of the genes (see reference 63 and Data Set S1C for a full list) reveals that gene families coding for enzymes that are involved in the metabolism of thiosulfate were far more abundant in the cone sediment samples than in the nearby vegetated soils, specifically, thiosulfate reductase (*phsA*, *phsB*, *phsC*) and thiosulfate sulfurtransferase (*glpE*) (Fig. 3). Other thiosulfate genes from the sulfur oxidation pathway, including *soxA*, *soxB*, *soxC*, *soxD*, *soxX*, *soxY*, and *soxZ*, thiosulfate dehydrogenase (*tsdA*, *tsdB*), and thiosulfate sulfurtransferase (*sseA*), were found in all metagenomes, though there were no significant differences between the two sample categories for any of these genes (Mann-Whitney U test, *P* > 0.05) (Fig. 3). Thiosulfate acts as an intermediate in metabolic pathways that reduce, disproportionate, or oxidize inorganic sulfur compounds (64), and thiosulfate is metabolized by almost all sulfur-metabolizing chemolithotrophs, including those from deep sea hydrothermal vent systems (65) and volcanoes (13). In the case of the surface sediments on HTHH, which we would expect to be well oxygenated, these genes are not likely associated with anaerobic processes of sulfur reduction and disproportionation. Rather, our results suggest that the oxidation of inorganic sulfur compounds is likely an important strategy used by microbes to survive on HTHH.

Photosynthetic cyanobacteria were not found in the cone sediment samples (Data Set S1B), and there was a corresponding absence of genes associated with oxygenic photosynthesis in the cone sediment metagenomes (Table S2; Data Set S1D). However, we did observe that genes associated with other forms of phototrophy were enriched in the cone samples (Fig. 3). In particular, the *pufM* and *pufL* genes were, on average, >3 times more abundant in the cone samples than in the vegetated soils (Fig. 3). The *pufML* gene family codes for the type II photosynthetic reaction center used by phototrophic organisms and anoxygenic photosynthesizers across a range of phyla (66), including some (like *Actinobacteria*) (Fig. 3) that were abundant in the cone samples. However, we only found the *pufML* genes in two of our recovered MAGs, members of the families *Beijerinckiaceae* and *Acetobaceraceae* within the phyla *Proteobacteria* and *Actinobacteria*, respectively (Fig. 3). While these putative phototrophs are not the dominant taxa in the cone samples, their presence suggests that anoxygenic photosynthesis, whereby sulfur is presumably used as an electron donor, may provide a competitive advantage in this environment.

We also found pronounced enrichment of gene-associated trace gas metabolism, namely, H_2_ and CO oxidation, in the cone sediment metagenomes. Many of the MAGs associated with the most abundant taxa recovered from our 16S rRNA gene sequencing effort (including *Chloroflexi* and *Ktedonobacteraceae*) do have the genetic capacity for trace gas metabolism (Fig. 3). More specifically, we identified genes for the catalytic subunits of group 1 (*NiFe*) hydrogenases, group 2 (*NiFe*) hydrogenases, and form 1 carbon monoxide (CO) dehydrogenases (*CoxL*) (67). Atmospheric H_2_ and CO oxidation are common strategies used by bacteria to sustain metabolic activity in resource-limited environments because these compounds are ubiquitous in the troposphere (68, 69). Organisms that use trace gas metabolism to sustain growth have been well described in Antarctic soils and other resource-limited systems (35, 68–70). While we acknowledge that the presence of genes does not necessarily indicate that these metabolisms are occurring *in situ*, such metabolic processes have been confirmed in recent volcanic ejecta (10). Likewise, trace gas oxidation has been shown to contribute significantly to microbial metabolism in older volcanic sediments, contributing to 6 to 10% of total respiratory activity 10 to 20 years after an eruption (67). Our findings highlight the potential importance of CO and H_2_ oxidation for microbes inhabiting even more recently deposited sediments. We hypothesize that trace gas oxidation may play a similar role to oxygenic photosynthesis in other primary successional environments, promoting microbial colonization of newly formed landmasses (11).

### Conclusions.

Our analyses of the microbes found on the island of HTHH suggest that microbes arrive soon after the formation of new volcanic islands. These microbial colonizers are not oxygenic photosynthesizers, as has been observed in other newly formed or exposed surfaces (like glacial forefields) where cyanobacteria typically dominate the earliest microbial communities (7, 11). Rather, the microbial colonizers on HTHH are more similar to taxa observed in terrestrial volcanic sediments and largely seem to be relying on trace gas and sulfur-based metabolisms to fuel microbial growth. Likewise, contrary to expectations, the initial colonizers are unlikely to be introduced to this newly formed landmass via dispersal from neighboring marine environments or vegetated soils. Rather, the microbes found on the surface of the young cone sediments appear to be derived from geothermally active environments, potentially including subsurface and/or subterranean sources. Volcanic activity may be seeding the earliest microbial colonizers, dispersing these organisms within and across systems, though it remains undetermined whether these early colonizing communities may have changed over the 3 years that elapsed between the formation of the island and when our samples were initially collected. We expect that if we had been able to continue to track the development of these communities over time, we would see a progression of microbial community change following models generated from other terrestrial volcanic systems, with chemolithotrophs decreasing in abundance as plants establish, organic matter accumulates, and soils develop over time (5, 6).

While we do not have direct field measurements of microbial growth and activity from these sediments, we assume that these taxa are indeed viable and active given that DNA from these organisms was recovered 3 to 4 years after the initial eruption. However, we do not know how long these organisms would persist and how these communities might continue to change over time as succession progresses. Unfortunately, the nearly complete destruction of the island of HTHH in January 2022 makes it impossible to revisit the site and conduct field measurements of metabolic activity on the subaerial cone, as has been done in previous studies (67, 70). These results need to be confirmed by performing *in situ* metabolic measurements in similar volcanic systems sampled soon after eruption or the next time a Surtseyan island emerges and persists.

## MATERIALS AND METHODS

### Sample collection.

Surface sediments (0 to 5 cm depth) were collected from Hunga Tonga Hunga Ha’apai (HTHH) by the scientists, students, and crew of the SSV *Robert C. Seamans* on 14 October 2018 and from 8 to 9 October 2019. A total of 32 sites were sampled (Fig. 1). Sediment was collected in sterile polyethylene bags using aseptic techniques. GPS coordinates, photographs of the soil surface, and other metadata were taken at the time of sample collection. Samples remained frozen at −20°C until they were processed at the University of Colorado Boulder (Boulder, CO, USA).

### Sediment characterization.

Sediment pH was determined for all samples according to the method described in reference 71. Specifically, 5 g of soil and 5 g of deionized (DI) water were placed in a 15-mL conical tube and shaken for 2 h at 200 rpm. Sediment pH was then measured with an Orion Star A211 benchtop pH meter (Thermo Fisher Scientific, Waltham, MA, USA). Other geochemical measurements were performed on freeze-dried and crushed aliquots of each sample. Total nitrogen content (TN) and total organic carbon (TOC) measurements for all samples were measured by the Arikaree Laboratory at the University of Colorado Boulder using a Shimadzu TOC-L/TNM-L TOC/TN analyzer. Additional geochemical analyses were performed on 25 of the samples by Activation Laboratories Ltd. (Ancaster, ON, Canada) with the composition of trace elements measured via lithium borate fusion inductively coupled plasma mass spectrometry (ICP-MS) and instrumental neutron activation analysis (INAA). Statistical tests to assess differences in the environmental and geochemical properties across sample categories were conducted using Mann-Whitney U tests for two groups or Kruskal-Wallis one-way analysis of variance tests with the *post hoc* Dunn’s multiple-comparison test for three or more.

### DNA extractions of sediment.

DNA was extracted from 0.5 g of each sample in a laminar flow hood using the Qiagen DNeasy PowerSoil kit (Qiagen, Germantown, MD, USA) following the manufacturer's recommendations. A total of 2 extraction blanks were included to test for any possible contamination introduced during the DNA extraction.

### Cultivation-independent microbial analyses via marker gene sequencing.

The DNA aliquots extracted from each of the 32 samples and their associated 2 extraction blanks were PCR amplified using a primer pair that targets the hypervariable V4 region of the archaeal and bacterial 16S rRNA (rRNA) gene (515F, 5′-GTGCCAGCMGCCGCGGTAA-3′, and 806-R, 5′-GGACTACHVGGGTWTCTAAT-3′) (72). This primer set included the appropriate Illumina adapters and unique 12-bp barcode sequences to permit multiplexed sequencing (73). Two no-template PCR blanks were included with each set of PCR amplifications. Amplifications were performed on a SimpliAmp thermal cycler (Thermo Fisher Scientific, Waltham, MA, USA) using Platinum II Hot-Start PCR master mix (2×) (Invitrogen, Carlsbad, CA, USA) in 25-μL reaction volumes. Cycling parameters consisted of an initial denaturation step at 94°C for 3 min, followed by 35 cycles of denaturation at 94°C (45 s), annealing at 50°C (60 s), extension at 70°C (90 s), and a final extension step at 72°C for 10 min.

The amplified products were cleaned and normalized to equimolar concentrations using SequalPrep normalization plates (Thermo Fisher Scientific, Carlsbad, CA, USA) and were sequenced on the Illumina MiSeq platform (Illumina, San Diego, CA, USA) using the V2 2 × 150-bp paired-end Illumina sequencing kit at the University of Colorado Boulder’s Next-Generation Sequencing Facility.

The 16S rRNA gene sequences were processed using the DADA2 pipeline v.3.8 (74). Sequences were quality filtered and clustered into exact sequence variants (ASVs), with taxonomy determined using a naive Bayesian classifier method (75) trained against the SILVA v.132 reference database (76, 77). A minimum bootstrapping threshold required to return a taxonomic classification of 50% similarity was used for analysis. The two extraction blanks yielded a total of 593 reads. None of the 11 ASVs that these reads were assigned to were found in any of the samples. The no-template PCR controls had reads associated with 37 ASVs, 6 of which were also found in other samples. However, reads associated with these ASVs were not common and only accounted for 0.04% of the reads from all extracted samples and were found in a maximum of 2 samples. Prior to downstream analyses, ASVs associated with chloroplast, mitochondria, or eukaryotes (137 ASVs total) and those unassigned to the phylum level (211 ASVs) were removed, as were ASVs with fewer than 10 reads across all samples. After this filtering, samples were included in downstream analysis if they met a threshold of 10,000 reads per sample. This left 27 samples that had sufficient prokaryotic 16S rRNA gene reads for downstream analyses with a mean number of reads per sample of 52,000 (range, 12,207 to 86,863).

### Microbial community analyses.

Community analyses of the sequenced soils were performed in R v.4.0.5 (78). Richness, the number of distinct prokaryotic ASVs out of 10,000 reads per sample, was calculated from the filtered 16S rRNA gene ASV tables using specnumber (R package vegan) (79). Jaccard distances were also calculated with vegan, using the function vegdist (method, jaccard). Plots of relative abundance were created using the R package mctoolsr (https://github.com/leffj/mctoolsr/), as were the nonmetric multidimensional scaling (NMDS) plots. Phylogenetic tree construction was performed with the 100 most abundant bacterial ASVs identified in the cone samples. Phylogenetic relatedness of the 100 ASVs was determined via maximum likelihood with RAxML v.8.0.0 (raxmlHPC -f a -m GTRGAMMA -p 12345 -x 12345 -number 100 [80]), including “*Candidatus* Cenarchaeum symbiosum” as the outgroup. Sequences were aligned using MUSCLE v.3.8.1551 (81), and the tree was visualized and annotated using iTOL v.6.3.2 (82).

To determine the percentage of taxa identified in the inland cone sediments that were from families that had been previously cultured, we used the Ribosomal Database Project (RDP)’s SeqMatch tool to search for the sequences of the top 100 ASVs recovered from the cone against the RDP v.11.5 database of 16S rRNA sequences filtered to include just those from isolated organisms (83).

We note that while samples were collected in 2 different years, we do not treat them as distinct sample categories. Comparisons between years could not be made because the specific locations sampled in 2018 were distinct from those sampled in 2019. However, identical methods were used to collect and process all samples, and we found no significant differences in the composition of bacterial and archaeal communities across the 2 years (permutational analysis of variance [PERMANOVA], *r* < 0.01).

### Comparisons against reference databases of potential source environments.

To shed light on the potential origin of the microbial diversity on the island, we examined the ASVs recovered from the inland cone sediments for bacterial taxa characteristic of the potential source environments. First, we calculated the percentage of reads associated with the 6 bacterial families that were found to be the most ubiquitous in a survey of the guts of 74 bird species (52). In the reference study of the 74 bird species, these 6 families were found in every single sample (52), yet we found they only make up a total of 1.31% of the total reads recovered from the inland cone samples. Next, we searched for the 10 most abundant bacterial orders found in seawater (51). These 10 bacterial orders made up a total of 65% of all reads recovered in a global survey of the microorganisms in seawater (51) but, in total, represented <0.05% of the reads in the inland samples. See Table S1 in the supplemental material for more information about the identity of these organisms.

To compare the microbial communities in the inland cone sediments to soil environments, we performed a similar search as described above for the most abundant bacterial phylotypes identified in a global survey of soil environments (see Table S1 and reference 54 for more detail). We then took the representative 16S rRNA gene sequences for each of the top 100 ASVs identified in the inland cone and vegetated samples and performed a BLASTN search at 100% identity against a reference database of 515 bacterial taxa that dominate the microbial communities in soil environments (53).

To compare the top ASV sequences against communities in hydrothermal vent environments, we reprocessed sequencing data downloaded from NCBI (BioProject accession no. PRJNA546572) from samples collected from the Brothers volcano complex (58) with the DADA2 pipeline following the methods described previously. The sequences of the top 100 ASVs identified in the cone sediments, and the vegetated samples were then searched against this data set following the same methods described previously for the soil reference database.

### Quantitative PCR.

To estimate how prokaryotic DNA concentrations vary across the sample set, we used quantitative PCR (qPCR) to measure bacterial 16S rRNA gene copy numbers in the 32 sediment samples, 2 corresponding extraction blanks, and 2 no-template controls. These analyses were performed on a Bio-Rad CFX Connect real-time system (Bio-Rad Laboratories, Hercules, CA, USA) using the same primers and soil DNA extracts used for sequencing. Reaction conditions and details followed methods described previously in reference 84. Reactions were performed in triplicate for each sample with the average of the readings being used for analysis. Standard curves were calculated using purified genomic DNA from Escherichia coli. Calculated copy number measurements for each sample are reported as the number of E. coli genome equivalents per gram soil^−1^.

### Metagenomic sequencing.

A total of 19 samples were selected for shotgun sequencing. These samples included all the samples collected from the inland cone (>30 m from the ocean) and from vegetated soils that predate the eruption from the bordering island of Hunga Tonga. With aliquots of the same DNA pools used for the amplicon sequencing, we generated metagenomic libraries with the Nextera DNA Flex library preparation kit (Illumina, San Diego, CA, USA). The manufacturer’s protocol was followed except that the number of PCR cycles was increased for low biomass samples following suggestions by Illumina tech support based on previous studies (85). Libraries were sequenced on an Illumina NextSeq 500 run using a high-output 300-cycle kit with paired-end chemistry at the University of Colorado Boulder’s Next-Generation Sequencing Facility.

The raw paired-end sequencing reads were interleaved using BBMap v.38.94 (https://sourceforge.net/projects/bbmap/); Illumina adapters were removed using Cutadapt v.2.1 (86) and were filtered based on sequence quality using Sickle v.1.33 (-q 20 -I 50) (https://github.com/najoshi/sickle). Two samples (HTHH_2019_5, HTHH_2019_15) had fewer than 100 reads remaining after this quality filtering and were not included in downstream analysis. For the remaining samples, we had an average of 46.8 million quality-filtered reads per sample (range, 10.7 million to 64.7 million reads). The filtered FASTQ sequences for these remaining samples were reformatted to FASTA using BBMap.

We used phyloFlash v.3.0 (87) on these filtered reads to verify that the metagenomic data were consistent with the taxonomic composition of the bacterial communities as inferred from the 16S rRNA gene amplicon data. We tested the strength and significance of this relationship with a Mantel test comparing the Bray-Curtis dissimilarity matrices of the amplicon and metagenomic data sets performed using the R package vegan and found that they were well correlated (Mantel test, *r* = 0.90, *P* < 0.001) (Fig. S5) (79).

### Targeted gene analysis.

Targeted analyses of 294 genes related to photosynthesis, trace gas metabolism, sulfur metabolism, and iron metabolism (Data Set S1D) were performed on the 23 trimmed and quality-filtered metagenomes. Sequence reads were searched against compilations of protein sequences from existing databases and repositories using the BLASTx function of DIAMOND v.2.0.11 with a query coverage of 80% (88). The sequences of the trace gas genes were downloaded from the Greening lab metabolic marker gene database v.1 (89), the sequences of the iron metabolism genes were from the FeGenie database (90), and the sequences of the sulfur metabolism genes were those from the National Center for Biotechnology Information (NCBI) reference sequence database as compiled for SCycDB (63). The photosynthesis genes used were compiled from references 66, 89, and 91. For more information regarding the specific genes categories investigated, see Data Set S1D. We note that some of the genes could be shared across multiple pathways (e.g., *dsrA*, *dsrB*, and *dsrC* are all involved in both sulfur reduction and oxidation [63]). Following the methods of reference 70, positive hits were considered those with an identity threshold >60% and a maximum E value threshold of 10^−10^. For each gene, abundances were normalized based on the abundance of 14 single-copy genes calculated using the program SingleM v.13.2 (https://github.com/wwood/singlem) and are presented as normalized reads per million (RPM).

To make predictions about potential metabolic pathways that are enriched in the inland cone sediments compared to the samples from the vegetated island, genes were grouped into larger functional categories based on references 63, 66, and 89 to 91. Abundances of these pathways in each of these samples were determined by summing the normalized RPM of all genes associated with a pathway or gene family. Pathways were considered enriched in the inland samples if the abundance was, on average, significantly higher in the inland samples than in the vegetated soils that predate the 2014–2015 eruption. Significance was determined using Mann-Whitney U tests as described above.

### Recovery and analysis of metagenomically assembled genomes.

To determine the organisms that are responsible for the gene categories identified through the targeted gene analysis, metagenome-assembled genomes (MAGs) were recovered following methods described in reference 68. In short, the sickle-trimmed sequences for 13 of the inland samples were assembled individually using metaSPAdes v.3.13.0 (92) with default parameters. Reads were mapped to assembled scaffolds using Bowtie2 v.2.4.5 (93), and abundance files were created and formatted with SAMtools v.1.14 (94) and anvi’o v.7.1 (95, 96). Scaffolds were then used to obtain genome-resolved bins with CONCOCT v.1.1.0 (97), MaxBin2 v.2.2.4 (98), and MetaBAT2 v.2.12.1 (99), and an optimized, dereplicated, nonredundant set of bins was identified and refined using the Dereplication, Aggregation, and Scoring Tool (DAS_Tool) v.1.1.2 (100). The quality of the dereplicated bins was determined using CheckM v.1.1.3 1 (101). Following the suggestions outlined in reference 101, bins that were greater than 70% complete and <10% contaminated were considered high quality and were used for downstream analyses. Following these methods, we recovered 156 high-quality MAGs from the 13 samples. Taxonomy was assigned to each of these MAGs with the GTDB-Tk v.2.1.0 classify workflow (102) based on the Genome Taxonomy Database (103), and open reading frames were predicted with Prodigal v.2.6.3 (104).

To identify which MAGs contain genes associated with the pathways we found to be elevated in abundance in samples from the inland cone (see above), the 48 genes associated with anoxygenic photosynthesis, carbon monoxide oxidation, hydrogen oxidation, sulfur disproportionation, and sulfur reduction were queried against the translated amino acid sequences of each MAG using the BLASTP function of DIAMOND v.2.0.11 with a query coverage of 80%, identity threshold of 60%, and maximum E value threshold of 10^−10^ (87).

### Data availability.

The raw metagenomic data have been deposited in the NCBI Sequence Read Archive under accession number PRJNA914229.
