# Supplementary figures and images for: The Early Microbial Colonizers of a Short-Lived Volcanic Island in the Kingdom of Tonga
Source: mBio. 2023 Jan 11;14(1):e03313-22. doi: 10.1128/mbio.03313-22 (PMC9972954; doi:10.1128/mbio.03313-22)

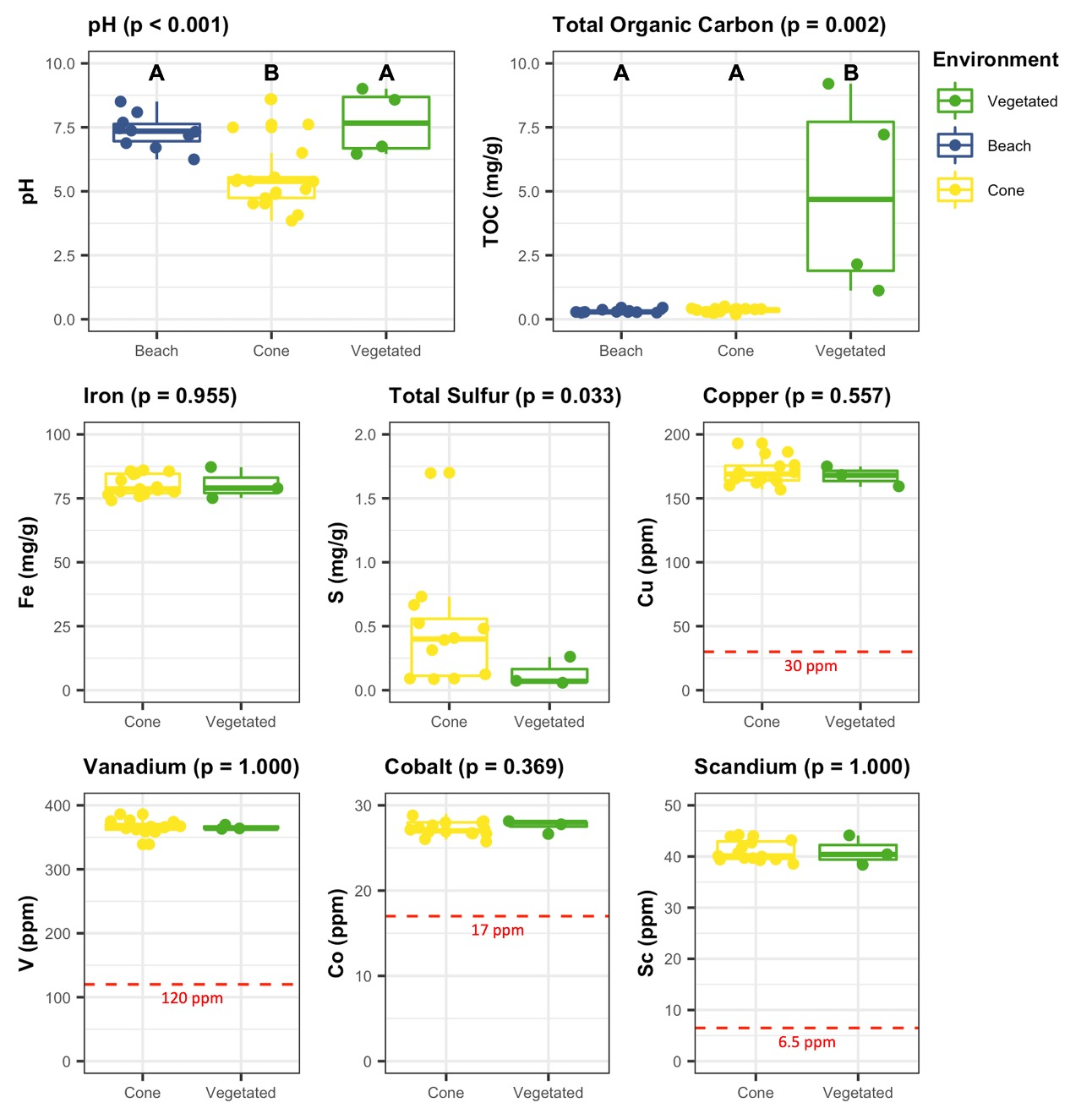

Supplement: FIG S1 [file mbio.03313-22-s0001.tif]

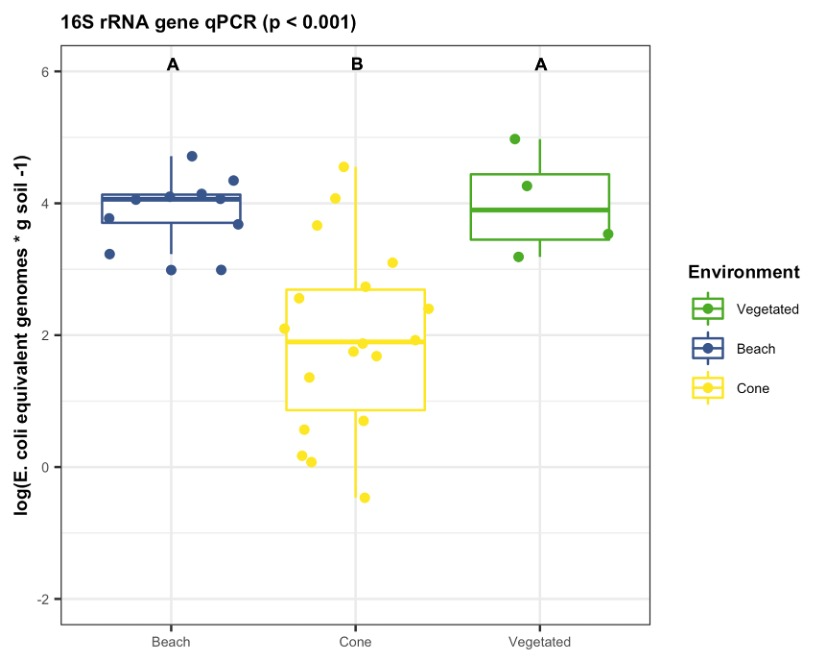

Supplement: FIG S2 [file mbio.03313-22-s0002.tif]

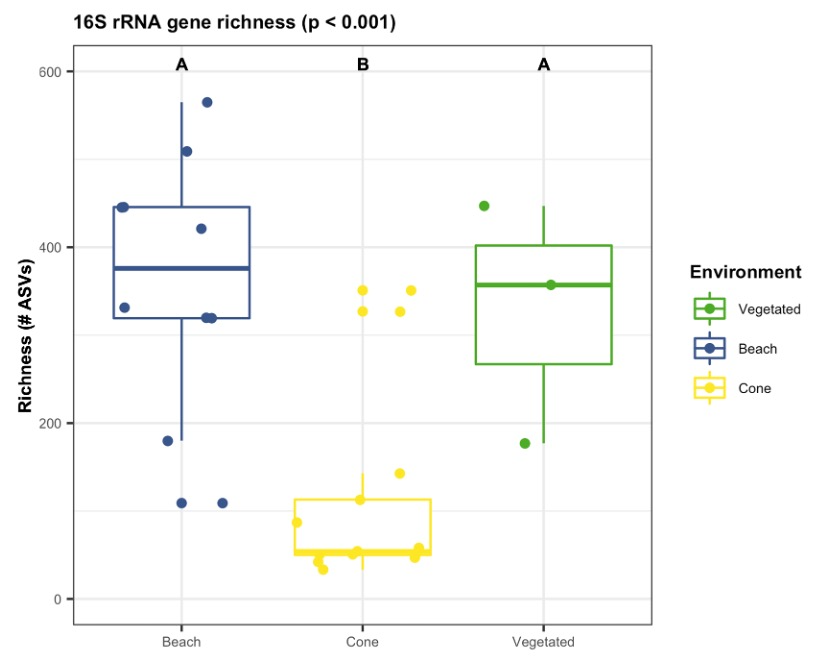

Supplement: FIG S3 [file mbio.03313-22-s0003.tif]

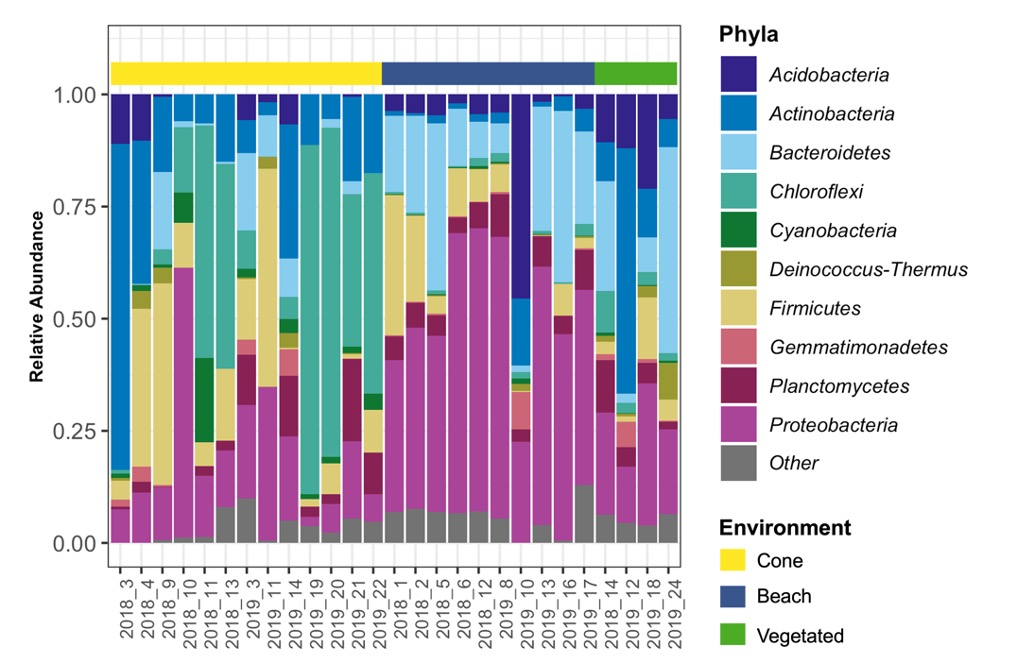

Supplement: FIG S4 [file mbio.03313-22-s0004.tif]

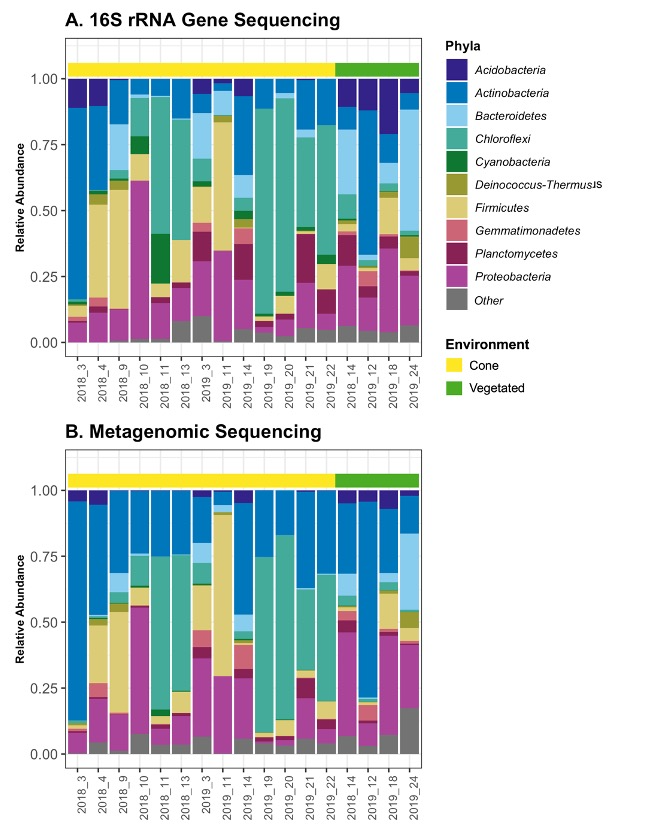

Supplement: FIG S5 [file mbio.03313-22-s0005.tif]

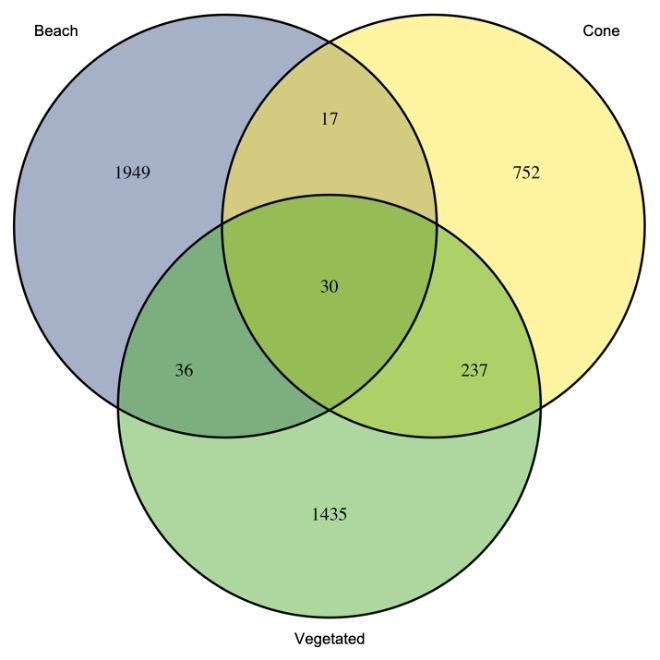

Supplement: FIG S6 [file mbio.03313-22-s0006.tif]

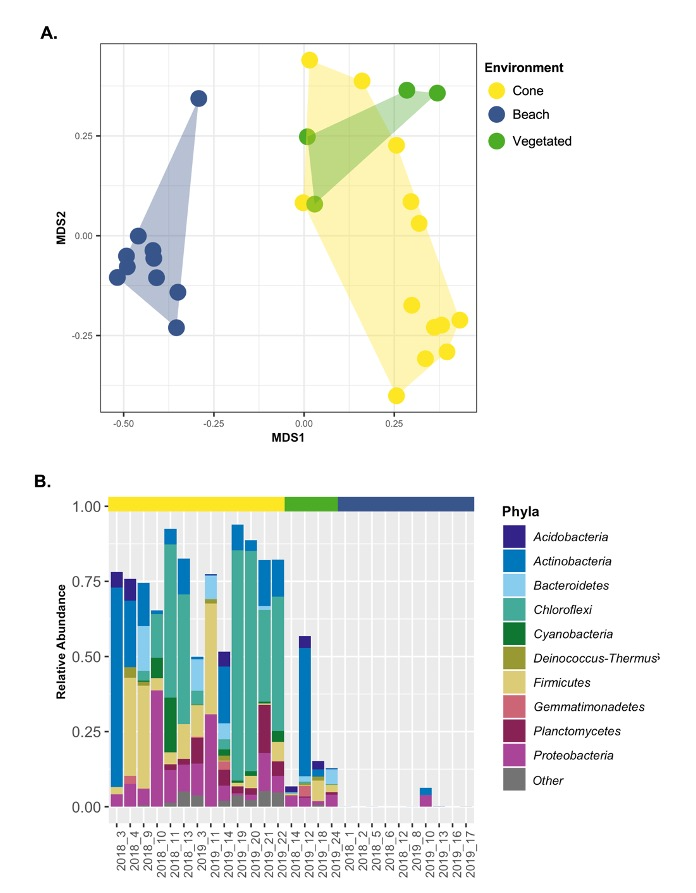

Supplement: FIG S7 [file mbio.03313-22-s0007.tif]

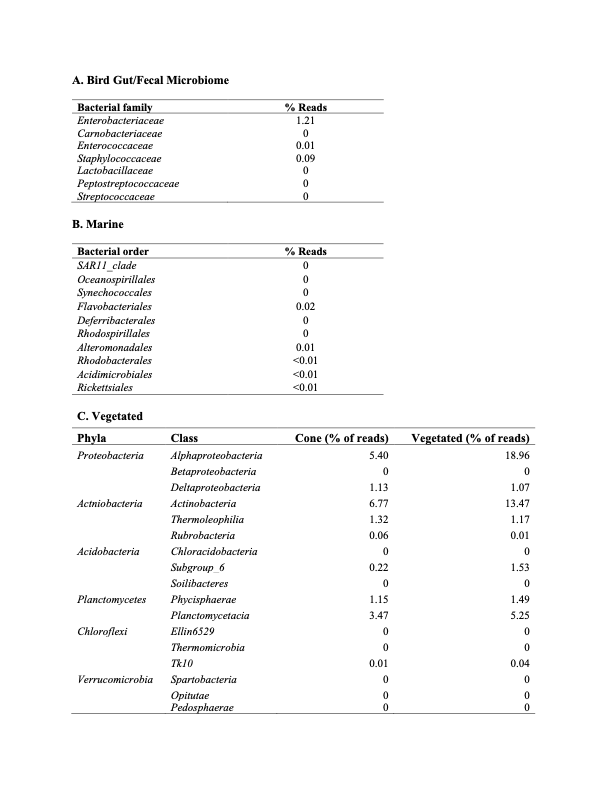

Supplement: TABLE S1 [file mbio.03313-22-s0008.tif]

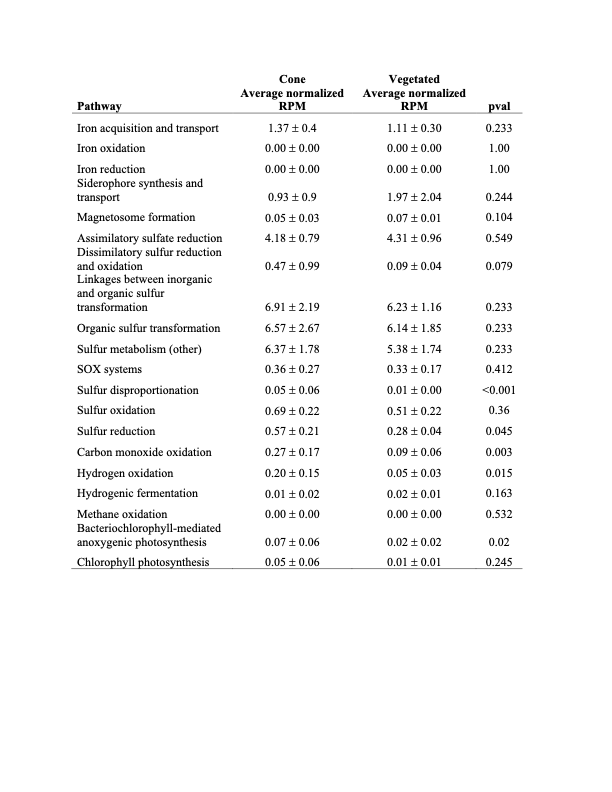

Supplement: TABLE S2 [file mbio.03313-22-s0009.tif]
